# A validated UPLC-MS/MS method for quantification of pyrotinib and population pharmacokinetic study of pyrotinib in HER2-positive breast cancer patients

**DOI:** 10.3389/fphar.2024.1432944

**Published:** 2024-09-20

**Authors:** Yunfang Zhu, Yuxiang Xu, Haopeng Zhao, Hongxin Qie, Xiaonan Gao, Jinglin Gao, Zhangying Feng, Jing Bai, Rui Feng, Mingxia Wang

**Affiliations:** ^1^ Department of Clinical Pharmacology, The Fourth Hospital of Hebei Medical University, Shijiazhuang, China; ^2^ Department of Pharmacy, The Fourth Hospital of Hebei Medical University, Shijiazhuang, China

**Keywords:** pyrotinib, tyrosine kinase inhibitor, population pharmacokinetics, HER2-positive breast cancer, NONMEM

## Abstract

**Objective:**

Pyrotinb has been approved for the treatment of HER2-positive advanced or metastatic breast cancer in China. However, the plasma concentration of pyrotinb in different patients varies greatly, and in the course of treatment, if patients have intolerable adverse reactions, the drug dosage will be reduced or even stopped. This study set out to establish an ultra-high performance liquid chromatography-tandem mass spectrometry (UPLC-MS/MS) for the determination of pyrotinb in human plasma, analyze the population pharmacokinetics (PPK) of pyrotinib and assess the influence of patient variables on PK of pyrotinib in patients with HER2 positive breast cancer.

**Method:**

An UPLC-MS/MS method was developed to measure pyrotinib in human plasma. Utilizing a gradient elution procedure and a Kinetex C18 column (2.1 mm × 100 mm, 1.7 μm), sample separation was accomplished in 5.5 min. Pyrotinb extraction via protein precipitation was used as a sample pre-treatment technique. In total, 50 patients provided 158 plasma samples, which were identified and used in the PPK investigation. The non-linear mixed-effects modeling (NONMEM) approach was used to assess the plasma concentrations and covariates information. For the final PPK model evaluation, external evaluation, non-parametric bootstrap, visual predictive check (VPC), and goodness-of-fit (GOF) were used.

**Results:**

The UPLC-MS/MS method for determining plasma concentration of pyrotinib in patients had good selectivity and linearity in the range of 1–1,000 ng/mL. Pyrotinib concentration profile in HER2-positive breast cancer patients was well described by a single-compartment PPK model with first-order absorption and elimination. The formulas for the final estimated values of overall parameters of CL/F and Vd/F and Ka are respectively: $CL/F\left(L/h\right)=88.8\times e^{\left(\text{TP}/67.2\right)\times 0.376}$ , $V/F\left(L\right)=3940$ , $KA\left(h^{-1}\right)=0.357FIXED$ . No dosage adjustment was advised, despite the possibility that the total protein levels could have a substantial impact on the apparent distribution volume of pyrotinib with limited magnitude.

**Conclusion:**

In this study, an UPLC-MS/MS method was established to determine the concentration of pyrotinib in human plasma. A population pharmacokinetic model of pyrotinib in HER2 positive breast cancer patients suggested that low serum total protein reduced the clearance rate of pyrotinib in patients. Clinical medical staff should pay attention to the liver function of patients with abnormal serum total protein and be alert to the occurrence of adverse drug reactions.

## 1 Introduction

Breast cancer (BC) is one of the malignant tumors with the highest morbidity and mortality in women today, and it is increasing year by year. According to molecular typing, BC is divided into four types: luminal A, luminal B, human epidermal growth factor receptor 2 (HER2)-positive and three-negative (Feng et al., 2018; Yeo and Guan, 2017). Among them, HER2-positive BC accounts for about 15%–20%, which has highly aggressive biological characteristics, high malignant degree (Loibl and Gianni, 2017), high recurrence rate and poor prognosis. At present, anti-HER2 targeted therapy mainly focuses on monoclonal antibodies, tyrosine kinase inhibitors (TKIs) and antibody-drug conjugates (ADCs). TKIs have the advantages of oral formulation, multiple targets and low toxicity. In addition, compared with monoclonal antibody drugs, small molecule TKIs can better cross the blood-brain barrier to achieve therapeutic effect (O’Sullivan et al., 2017; Gelmon et al., 2015; Awada et al., 2016).

Pyrotinib is an oral and irreversible TKI independently developed in China. In August 2018, this drug has been approved by the China National Medical Products Administration (CFDA) for the treatment of patients with HER2-positive advanced or metastatic breast cancer who had previously received anthracycline or taxane chemotherapy (Blair, 2018). Guan et al. (2023) summarized and analyzed individual patient-level data from three clinical trials (Phase I clinical trial: NCT02361112, Phase II clinical trial: NCT02422199, Phase III clinical trial: NCT03080805). Compared with patients treated with lapatinib + capecitabine (L + C), patients treated with pyrotinib + capecitabine (P + C) had significantly longer PFS (22.0 months vs*.* 6.9 months, *p* < 0.001) and OS (59.9 months vs*.* 31.2 months, *P *= 0.033). PFS was significantly longer in the P + C cohort than in the L + C cohort, regardless of whether patients had received trastuzumab previously (prior trastuzumab: 16.5 months vs*.* 6.9 months, *p* < 0.001; No prior trastuzumab: 27.5 months vs*.* 5.5 months, *P* = 0.004). These findings suggest that pyrotinib has superior efficacy as a treatment option for HER2-positive breast cancer.

The common adverse reactions of pyrotinib include diarrhea, hand-foot syndrome, AST/ALT elevation, etc. diarrhea is the most common and high incidence of adverse reactions. The results of a study on adverse reactions of targeted drugs for HER2-positive breast cancer (Fang et al., 2022) showed that all patients had varying degrees of diarrhea from 1 to 7 days after the first dose (2.76 ± 2.17 days). The duration of diarrhea was less than 1 week in 4 cases (8.7%), lasted from 1 week to 1 month in 19 cases (41.3%), and more than 3 months in 30.4%. The incidence of grade 3–4 diarrhea caused by pyrotinib reached as high as 80%. Diarrhea is an important factor hindering the absorption of drugs in the gastrointestinal tract (Effinger et al., 2019). In clinical practice, drug reducing or even withdrawal due to different degrees of diarrhea has become one of the important reasons affecting the subsequent treatment. Montmorillonite powder is one of the symptomatic drugs used for diarrhea caused by pyrotinib in clinical practice. Wen et al. (2021) showed that the combination of montserillonite powder in the treatment of diarrhea had a significant effect on the bioavailability of pyrotinib, and the distribution volume of pyrotinib could be significantly affected by patient age and serum total protein level.

Phase I trial data has indicated that large accumulation of pyrotinib was not observed with repeated daily dosing (Li et al., 2019). The steady-state apparent volume of distribution (Vd/F) and clearance (CL/F) of patients receiving 80–400 mg of pyrotinibranged from 2,570 ± 39.9 L to 3,820 ± 55.7 L and 149 ± 25.8 L/h to 213 ± 66.8 L/h, respectively (Ma et al., 2017). During elimination, pyrotinib is mainly metabolized by hepatic cytochrome P450 (CYP) 3A4 (>75%) and is mainly excreted in feces (>90.9%) (Meng et al., 2019; Zhu et al., 2016). Of interest is the high variability among subjects in phase I clinical trials after administration of pyrotinib. The coefficients of variation of AUC_0–24h_ and C_max_ at steady state varied from 25% to 110% and 32.8%–91%, respectively (Li et al., 2019; Ma et al., 2017). Therefore, it is essential to explore the potential influencing factors of pyrotinib PK in the patient population for individualized regimen administration and optimization of clinical results.

At present, there are few reports about the detection of plasma concentration of pyrotinib. Meng et al. (2019) and Zhu et al. (2016) analyzed the pharmacokinetics of pyrotinib in human body by radioactive liquid chromatography and ultra-high performance liquid chromatography-quadrupole-time-of-flight mass spectrometry (UPLC-Q-TOF-MS) respectively. In addition, Chai et al. (2021) used UPLC-MS/MS to simultaneously determine pyrotinib and its metabolite, pyrotinib-lactam, in rat plasma and applied it to the pharmacokinetic study of pyrotinib and its metabolite. Among the above methods, radioactive liquid chromatography is not suitable for routine development of clinical blood drug concentration monitoring. UPLC-Q-TOF-MS method needs expensive equipment and low penetration rate, so it is difficult to meet the needs of clinical blood drug concentration monitoring. Liquid chromatography-tandem mass spectrometry (LC-MS/MS) is the best choice to detect the plasma concentration of small molecule targeted drugs at home and abroad at present, which has the advantages of wide application range, high detection sensitivity, strong specificity and fast analysis speed.

In recent years, more and more attention has been paid to tyrosine kinase inhibitors in PPK. Although PPK modeling studies (Wen et al., 2021) of pyrotinib based on data from two phase I clinical trials (NCT01937689 and NCT02361112) have been reported, there is a huge difference between the study population and the real-world application. During the real world treatment, the dose would be reduced or discontinued when the intolerable adverse reactions occurred. Therefore, compared with clinical trials, the dosage of some patients in the real world is dynamic. It is necessary to conduct PPK studies on real-world sample data. The purpose of this study is to perform PPK modeling in HER2-positive breast cancer patients to reflect the clinical use of pyrotinib and evaluate the impact factors, such as patient demographic information, pathophysiological and pharmacotherapeutic characteristics on the PK of pyrotinib in clinical application.

Therefore, we aimed to establish a rapid, simple, and sensitive UPLC-MS/MS method for the determination of pyrotinib concentration in human plasma, and further apply it for the PPK research. This study is the first PPK study of pyrotinib applied in the real world, in order to provide reference for clinical individualized administration of pyrotinib.

## 2 Materials and methods

### 2.1 Chemicals materials

Pyrotinib (purity >95%; 6,124,170,301) and apatinib (purity> 99%; 668,160,504) were both gifted from Jiangsu Hengrui Pharmaceuticals Co., Ltd. (Jiangsu, China). HPLC-grade methanol and acetonitrile were purchased from Fisher Scientific (Waltham, MA, United States). Formic acid and ammonium acetate were obtained from Mreda Technology Inc. (Dallas, TX, United States). Ultrapure water was provided by the WatsonCompany (Guangzhou, China).

### 2.2 Instruments

An ExionLC™ analytical (UPLC) system (AB Sciex, United States) with an AD controller, binary pump, and degasser was used to perform chromatographic analysis. An electrospray ionization (ESI) source operating in the positive ion mode was fitted to an AB Sciex Triple Quad 5,500 mass spectrometer (5500 Q-trap; Applied Biosystems Inc., United States) for use in MS analysis.

### 2.3 Solutions preparation

In order to create a 1 mg/mL standard stock solution, 10 mg of pyrotinib was added to a 10 mL volumetric flask and dissolved with methanol. The same procedure was used to create apatinib (internal standard, IS) stock solution (1 mg/mL). Working solutions of 10, 50, 100, 500, 1,000, 2,000, 5,000 ng/mL, and 10 μg/mL for pyrotinib were obtained by diluting a stock solution of 1 mg/mL of the drug with acetonitrile/water (1:1, v/v). A series of calibration standard sample solutions of pyrotinib with final concentrations of 1, 5, 10, 50, 100, 200, 500, and 1,000 ng/mL were generated by adding 10 μL of working solution of pyrotinib to 90 μL of drug-free human plasma. In the same manner, the middle quality control (MQC; 80 ng/mL), high quality control (HQC; 800 ng/mL), low quality control (LQC; 2 ng/mL), and lower limit of quantitation (LLOQ; 1 ng/mL) were generated. Methanol was used to dilute the apatinib stock solution to create an IS working solution with a concentration of 10 ng/mL.

### 2.4 Plasma sample treatment

100 μL of drug-containing human plasma and 400 μL of precipitant containing 10 ng/mL of IS were combined in 1.5 mL centrifuge tubes, vortexed for 3 min, and centrifuged at 13,000 × g for 5 min. Subsequently, 200 μL of water was added to 100 μL of supernatant, vortexed for a minute, then centrifuged for 3 min at a speed of 130,00× g. Ultimately, the UPLC-MS/MS apparatus was injected with 5 μL of the supernatant for analysis.

### 2.5 Analytical conditions

The temperature was kept at 45°C during the chromatographic analysis, which was carried out on a Kinetex C18 column (2.1 mm × 100 mm, 1.7 μm; Phenomenex, United States). With a gradient elution procedure: 0∼0.3 min, 35%B; 0.3∼0.8 min, 35%→82%B; 0.8–2.6 min, 82%B; 2.6∼3 min, 82%→35%B; 3∼5 min, 35%B. The mobile phase was composed of 0.1% formic acid and 10 mM ammonium formate water (A) and acetonitrile (B). An injection of 5 μL of sample at a flow rate of 0.3 mL/min was made into the UPLC-MS/MS system with the autosampler set at 4°C.

**FIGURE 1:**
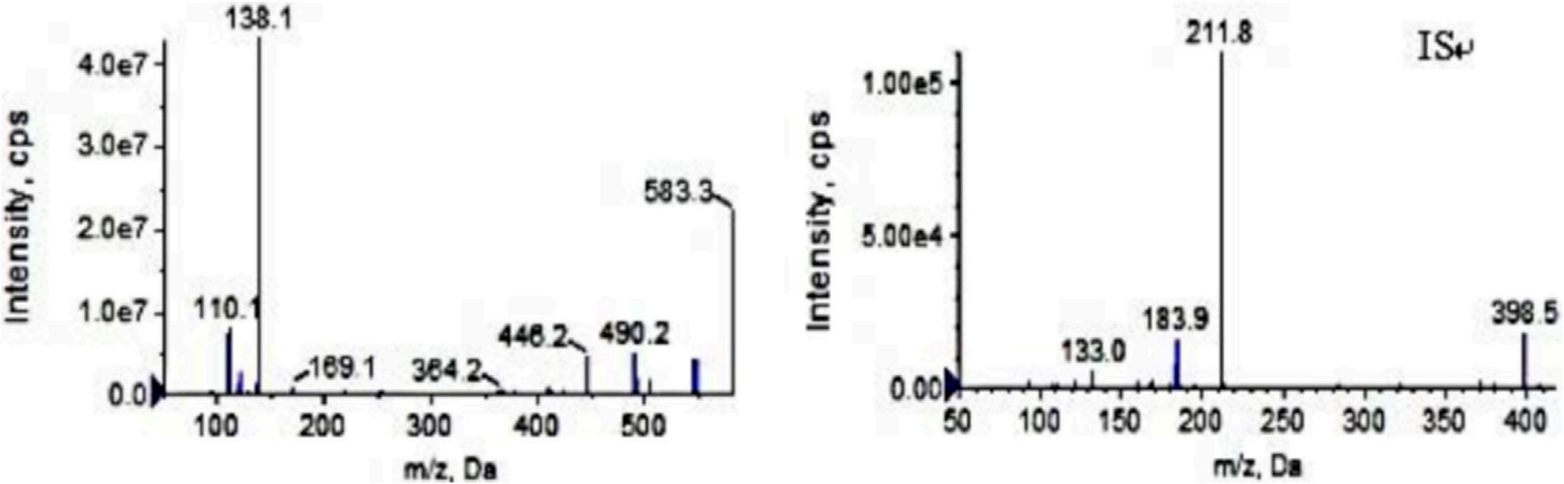
The product ion plots of pyrotinib (left) andapatinib (IS, right).

Ion source: electrospray ion source; Ion detection mode: positive ion scanning in MRM mode; Air curtain pressure: 35 psi, collision gas pressure: Medium, atomizing gas pressure: 40 psi, auxiliary heating gas pressure: 50 psi, ion source voltage: 5,500 V, ion source temperature: 500°C. The following mass transition pairs were observed by multiple reaction monitoring (MRM): m/z 583.3→138.2 for pyrotinib and m/z 398.2→212.0 for IS. Declustering potentials for pyrotinib and IS were 85 V and 70V, respectively, and their collision energies were 36 eV and 41 eV, respectively. Figure 1 displays the secondary mass spectrograms of the IS and pyrotinib.

### 2.6 Method validation

The accuracy of the UPLC-MS/MS method for determining pirotinib in human plasma was confirmed by following the guidelines provided by the US Food and Drug Administration and China Pharmacopoeia (FDA, 2018; Xu et al., 2019; Tang et al., 2021).

#### 2.6.1 Specificity and selectivity

By comparing the chromatograms of blank human plasma samples from various sources, blank human plasma spiked with pyrotinib and IS, and human plasma samples following pyrotinib treatment, the specificity of the UPLC-MS/MS approach was assessed. For the analytes at the LLOQ, the corresponding reaction should be less than 20%, and for the IS, it should be 5%.

#### 2.6.2 Calibration curve, linearity, lower limit of quantitation and limit of detection

Using linear least squares and 1/x^2^ weighted regression, the peak area ratio of pyrotinib (1, 5, 10, 50, 100, 200, 500, and 1,000 ng/mL) to the IS was plotted against the nominal concentrations to produce the calibration curves. The standard curve’s regression coefficient (r) ought to be higher than 0.99. The back-calculated concentrations at the LLOQ should be within ±20% of the nominal values, but not more than 15% of them. The lowest concentration on the calibration curve with S/N > 10 was designated as the LLOQ. The limit of detection (LOD) was defined as S/N ratios of 3.

#### 2.6.3 Precision and accuracy

By identifying the QC samples of pyrotinib at four concentration levels in five repetitions on the same day and on more than 2 days, respectively, the intra- and inter-batch precision and accuracy were assessed. The percentage of the measured concentration to the theoretical concentration was used to calculate accuracy, and the relative standard deviation (RSD%) was used to describe precision.

#### 2.6.4 Carryover

Analyzing a blank sample following the injection of the highest calibration standard sample allowed for the evaluation of carryover. The analyte peak area in the blank sample should not be more than 5% of the internal standard or 20% of the LLOQ.

#### 2.6.5 Matrix effect and recovery

The IS-normalized matrix factor (MF) at the LQC and HQC concentration levels was used to assess the matrix effect, which is the differential ionization of analyte(s) due to matrix components present in the biological samples. The ratio of the peak area with the matrix present (blank matrix spiked after extraction) to the peak area without the matrix present (neat aqueous samples) was used to compute the matrix factor (MF). The MF of pyrotinib was divided by the MF of IS to obtain the IS-normalized MF. Lastly, comparisons were made between the RSDs (%) of the IS-normalized MF. The ratio between the peak area extracted with the matrix (blank matrix spiked prior to extraction) and the peak area in the presence of the matrix (blankmatrix spiked post-extraction) was used to calculate recovery.

#### 2.6.6 Stability

The stability of plasma samples was examined at LLQC, LQC, MQC, and HQC under four distinct storage scenarios: 4 hours at ambient temperature, 24 h at 4°C in an auto-sampler tray processing sample, three cycles of freeze-thaw (−80°C), and 4 months at −80°C. 90 μL of blank plasma was placed in a 1.5 mL centrifuge tube, and 10 μL of pyrotinib QC working solution with LLQC, LQC, MQC, and HQC (10, 20, 800, 8,000 ng/mL) were added respectively to prepare QC blood samples, which were vortex-mixed for 1 min and centrifuged for 30 s. The precision and accuracy of stability were evaluated by determining the QC samples of pyrotinib at four concentration levels in five replicates.

## 3 Study design and patients

### 3.1 Patients and datasets

#### 3.1.1 Patients

A total of 50 patients with HER2-positive breast cancer who received pyrotinib in the Fourth Hospital of Hebei Medical University from November 2020 to November 2023 were enrolled in this study. The study was conducted in accordance with principles in the Declaration of Helsinki, and was approved by the ethics committee of the Fourth Hospital of Hebei Medical University, Shijiazhuang, China (No.2020032).

Inclusion Criteria: HER2-positive breast cancer; regular oral pyrotinib (400 mg, 320 mg, and 240 mg); age ranged from 18 to 80 years. Exclusion Criteria: demographic and laboratory data missing; incomplete medical record information; treatment discontinuedfor 1 month or more.

#### 3.1.2 Administration protocol and blood sample processing

Patients took pyrotinib maleate tablets (400 mg, 320 mg and 240 mg) orally 30 min after meals daily. Clinical plasma samples were collected from patients by opportunistic blood sampling. The relevant information of patients (such as demographics, pathophysiology, etc.) was collected through follow-up and consulting the HIS system of medical records from the Fourth Hospital of Hebei Medical University.

Pyrotinib plasma concentrations were measured using the validated UPLC-MS/MS method.

#### 3.1.3 Data handling

Patients with a pyrotinib plasma concentration result of ≥1 and an acceptable dose of ≥ 1 were included in the PK analysis. Analysis of concentrations below the LLOQ was omitted. Data outside the conditional weighted residual errors (CWRES) range of −6 to 6 were deemed possible outliers by exploratory analysis and were not included in the modeling analysis. If a covariate was absent in more than 20% of the patients, it was eliminated.

### 3.2 Population pharmacokinetic analysis

Using first-order conditional estimation with the η–ε interaction (FOCE-I) method, a nonlinear mixed-effect modeling (NONMEM) approach was used for building PPK models. This was done using the NONMEM program (version 7.5.0, ICON Development Solutions, Ellicott City, MD, United States). Visual prediction checks, covariate screening, and bootstrap analyses were carried out using Perl-speaks-NONMEM (PsN, version 5.2.6, Department of Pharmaceutical Biosciences, Uppsala University, Sweden). The R package “Xpose” (version 4.5.3, Department of Pharmaceutical Biosciences, Uppsala University, Sweden), R (version 4.2.1, R Foundation for Statistical Computing, Vienna, Austria), and SPSS (version 21.0) were utilized for data processing and graphical analysis.

#### 3.2.1 Base model

One- and two-compartment models were chosen as potential structural models based on graphical exploratory research. The structural pharmacokinetic parameter between-subject-variability (BSV) was included. Based on an exponential model (Equation 1), BSV was used as follows:

$$P_{i}=P_{pop}\times^{e(\eta_{i})}$$ (1)

where η_i_ represents the relationship between individual random effects for individual i, which is assumed to be normally distributed with a mean of zero and variance of ω2. P_i_ stands for the individual parameter estimate for individual i; P_pop_ stands for the typical population parameter estimate.

The residual unexplained variability (RUV) was modeled using one of three methods: proportional (Equation 2), additive (Equation 3), or a combination of both (Equation 4).

$$Y_{\text{ij}}=IPRED_{\text{ij}}\times \left(1+\varepsilon_{p,ij}\right)$$ (2)

$$Y_{\text{ij}}=\text{IPRE}D_{\text{ij}}+\varepsilon_{p,ij}$$ (3)

$$Y_{ij}=IPRED_{ij}\times \left(1+\varepsilon_{p,ij}\right)+\varepsilon_{a,ij}$$ (4)

where IPRED_ij_ is the individual predicted concentration; Y_ij_ is the observed concentration for the individual i at time tj; ε_p,ij_ denotes the proportional error component; and ε_a,ij_ is the additive error component. It was assumed that residual error had a normal distribution with a variance of σ2 and a mean of zero.

The base model was built according to the objective function value (OFV), visual assessment of goodness-of-fit (GOF) plots, and the accuracy of parameter estimates.

#### 3.2.2 Covariate model

Based on physiological and clinical plausibility, potential PK variables included: age, height (HT),weight (WT), body mass index (BMI), serum sodium (Na), serum kalium(K), albumin (ALB), globulin (GLB), total protein (TP), aspartate aminotransferase (AST), alanine aminotransferase (ALT), total bilirubin (TBIL), Direct Bilirubin (DBIL), Indirect Bilirubin (IBIL), serum creatinine (SCr), occurrence of diarrhea, combined medications and immunohistochemical results.

In the initial investigation, statistical research was done on covariate correlations. For additional analysis, one of the highly associated factors was kept. Plotting empirical Bayes estimates of individual parameters versus covariates allowed us to investigate the effects of variables on pharmacokinetic parameters. Subsequently, covariates that were recognized as possibly impacting pharmacokinetic parameters underwent formal testing using a stepwise forward inclusion and backward elimination procedure.

One potential covariate at a time was introduced to the base model throughout the forward inclusion procedure, and each additional component necessitated a considerable drop in the objective function value (>3.84, *p* < 0.05). Upon include all significant factors, a comprehensive model was produced. One covariate at a time from the entire model was eliminated during the backward elimination process, and a substantial change in the objective function value (>10.83, *p* < 0.001) after the deletion was the condition for keeping a covariate. Covariates that satisfied the previously stated statistical requirements were included in the final model and kept because of their clinical significance. Covariates that had an impact on the parameter of greater than 20% were considered to have clinical relevance.

Both a power function (Equation 7) and an exponential model (Equation 6) evaluated continuous covariates, whereas (Equation 8) tested categorical covariates:

$$\theta_{i}=\theta_{1}+\theta_{2}\times \left({\text{cov}}_{i}÷{\text{cov}}_{median}\right)$$ (5)

$$\theta_{i}=\theta_{1}\times {\left({\text{cov}}_{i}÷{\text{cov}}_{median}\right)}^{\theta_{2}}$$ (6)

$$\theta_{i}=\theta_{1}\times {\theta_{2}}^{\left({\text{cov}}_{i}÷{\text{cov}}_{median}\right)}$$ (7)

$$\theta_{i}=\theta_{1}\times {\theta_{2}}^{{\text{cov}}_{i}}$$ (8)

where the parameters for the person i are described by θ_i_ and cov_i_, respectively, as the covariate value. For every covariate, the median value is called covmedian. The pharmacokinetic parameter’s usual value at the median covariate value is represented by θ_1_+θ_2_, θ_1_, and θ_2_ in Equations 5–7, in that order. For categorical variables, cov_i_ in Equation 8 equals 0 or 1.

Covariate analysis was also conducted using a full model approach.

#### 3.2.3 Model evaluation

GOF plots were utilized to assess the model’s fitness. The observed values versus population projected values, observed values versus individual predicted values, CWRES versus population predicted values, and CWRES versus time were all assessed using scatter plots. For the purpose of evaluating the model, nonparametric bootstrap resampling (n = 1,000) was also used. Through the application of the final model to 1,000 bootstrapped datasets, each parameter was estimated many times. The estimates of the final model were compared with the 2.5th, 97.5th, and median popPK parameter estimates from the resampled datasets.

For simulation-based model assessment, the visual predictive check (VPC) method was employed to account for variations in independent variablest (Bergstrand et al., 2011). Using the final model’s parameters, 1,000 datasets were simulated in order to apply the VPC technique. The 5th and 95th percentiles as well as the median from the observed data were compared to the corresponding percentiles and median from the simulated data. An evaluation was conducted on the concordance of central tendency and variability between the simulated and observed data.

## 4 Results

### 4.1 Validation of methodology

#### 4.1.1 Specificity and selectivity

**FIGURE 2:**
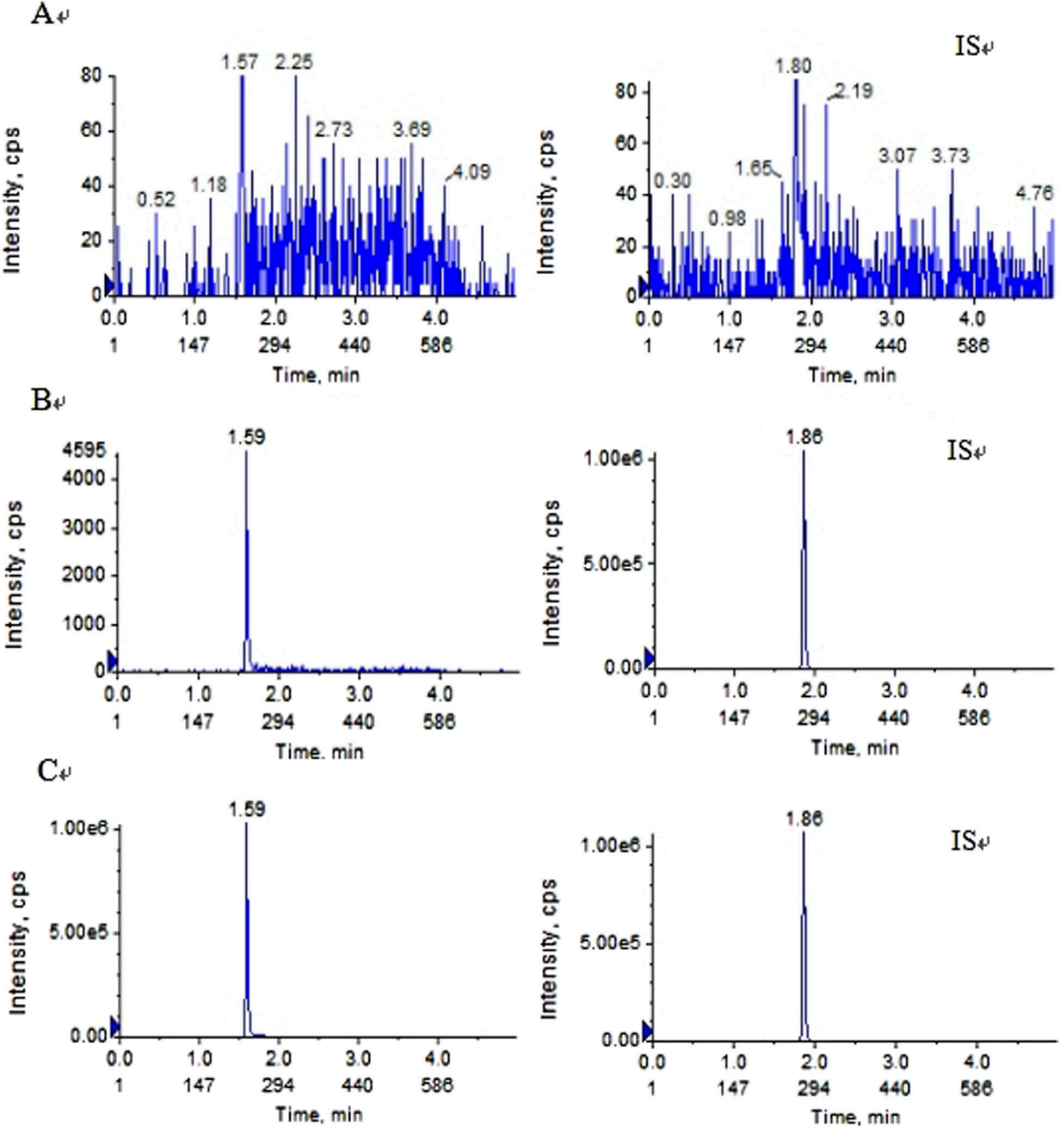
Typical MRM chromatograms of pyrotinib (left) and IS (right):**(A)** blankplasma sample **(B)** blank plasma sample spiked with pyrotinib at 1 ng/mL and IS at 10 ng/mL; and **(C)** plasma determination in patients with oral pyrotinib.

Pyrotinib and IS were fully isolated in the above experimental setup, and endogenous chemicals had no effect on detection. Figure 2 displays representative chromatograms. Pyrotinib and the IS had retention times of 1.59 and 1.86 min, respectively. The fact that no notable interferences were seen during the IS and pyrotinib retention periods suggests that this approach was adequately targeted.

#### 4.1.2 Calibration curve, linearity and lower limit of quantitation

At the pyrotinib concentration range of 1–1,000 ng/mL, the typical regressionequation of pyrotinib was y = 0.00375–6.31213e^−4^ (r = 0.9998), which exhibited excellent linearity. The LLOQ was 1 ng/mL with an accuracy of 80%–120%, and the RSD values representing precision were less than 15%. The LOD was 0.1 ng/mL.

#### 4.1.3 Precision and accuracy

The precision of the LLOQ and QC samples for intra- and inter-batch complied with Chinese and FDA Pharmacopoeia criteria when compared to the theoretical concentration, and all of these results were acceptable (Table 1). For pyrotinib, the intra-batch precision and accuracy were 1.95%–3.85% and 101.29%–108.46%, respectively, in five samples at each QC level. For every QC level, the inter-batch accuracy and precision were assessed in triplicate and were 6.23%–9.25% and 99.52%–103.79%, respectively.

**TABLE 1:** Intra-day and inter-day precision and accuracy for pyrotinib of quality control samples.

QC (Nominalconc) (ng/mL)	Intra-batch (n = 5)			Inter-batch (n = 15)		
	Measured conc Mean ± SD (ng/mL)	RSD%	Accuracy (%)	Measured conc Mean ± SD (ng/mL)	RSD%	Accuracy (%)
1	1.01 ± 0.04	3.48	101.29	1.04 ± 0.09	8.81	103.79
2	2.08 ± 0.08	3.85	103.90	2.02 ± 0.13	6.23	101.18
80	86.36 ± 1.68	1.95	107.95	79.62 ± 5.76	7.23	99.52
800	867.7 ± 27.56	3.18	108.46	803.06 ± 74.32	9.25	100.38

#### 4.1.4 Carryover

The analyte peak area in the blank sample satisfied the criteria for clinical sample determination, measuring 3.56% of the LLOQ and 0.01% of the IS in each valid batch.

#### 4.1.5 Matrix effect and recovery

The findings demonstrated that, at pyrotinib doses of LQC (2 ng/mL) and HQC (800 ng/mL), the IS-normalized MFs (RSD%) were 89.56% (7.42%) and 89.79% (9.46%), respectively. At LQC (2 ng/mL), MQC (80 ng/mL), and HQC (800 ng/mL), the recovery rates of pyrotinib were found to be 79.80%, 78.91%, and 82.14%, respectively. The IS recovered at a rate of 105%.

#### 4.1.6 Stability

Under the previously mentioned parameters, pyrotinib in human plasma was found to be stable. All stability results are compiled in Table 2.

**TABLE 2:** Stability of pyrotinib in human plasma.

Stability experiment	Nominal conc. (ng/mL)	Measured conc. Mean ± SD (ng/mL)	Accuracy (%)	RSD (%)
Autosampler for 24 h	1	0.89	88.77	4.51
	2	1.96	98.02	2.98
	80	76.53	95.67	1.90
	800	752.44	94.05	4.81
Room temperature for 4 h	1	0.87	86.54	7.97
	2	1.85	92.25	7.23
	80	79.18	98.97	2.22
	800	834.44	104.30	2.37
3 freeze-thaw cycles at −80°C	1	1.00	100.20	4.58
	2	1.98	98.79	1.14
	80	84.87	106.09	1.88
	800	819.08	102.39	7.50
−80°C for 4 months	1	0.99	99.17	6.34
	2	2.05	102.56	5.78
	80	84.28	105.35	4.66
	800	845.12	105.64	2.68

### 4.2 Patients and datasets

#### 4.2.1 Patient characteristics

A total of 50 patients with HER2-positive breast cancer were included in the study, and a total of 158 concentration points were used as the modeling group. All patient details including demographic information, laboratory biochemical measures, and other relevant information are provided in Table 3.

**TABLE 3:** Baseline demographics characteristics and clinical laboratory measurements of patients included in the model development.

Baseline information	Modeling group
Number of patients	50
Number of concentrations	158
AGE (year)	60.5 ± 11.9 (53 34–68)
HT (cm)	73.4 ± 24.4 (158,150–166)
WT (kg)	163.7 ± 23.0 (61.5 52.5–86)
BMI(kg/m2)	24.0 ± 2.6 (24.2 17.0–31.6)
Na (mmol/L)	22.7 ± 14.1 (138.6 123.2–145.2)
K (mmol/L)	25.4 ± 13.2 (4.1 2.27–5.47)
ALB (g/L)	66.3 ± 6.1 (41.4 12.2–51.5)
GLB (g/L)	40.0 ± 3.8 (26.1 15.1–38.1)
TP (g/L)	26.6 ± 5.0 (67.2 49.0–80.7)
AST (U/L)	94.9 ± 30.3 (19.8 11.8–327.1)
ALT (U/L)	5.9 ± 2.4 (16.7 6.6–193.6)
TBIL (μmol/L)	8.7 ± 23.9 (8.0 3.3–77.0)
DBIL (μmol/L)	232.9 ± 86.8 (2.2 0.1–49.9)
IBIL (μmol/L)	10.1 ± 5.3 (5.3 0.3–27.1)
SCR (μmol/L)	3.5 ± 2.1 (60.5 30.8–419.0)
DRH (yes/no)	24 (48%)/26 (52%)
MP (yes/no)	9 (18%)/41 (82%)
LC (yes/no)	15 (30%)/35 (70)
ER (positive/negative)	28 (56%)/18 (36%)
PR (positive/negative)	25 (50%)/21 (42%)

Data for continuous variables are presented as means ± standard deviations. Data for categorical variables were expressed as quantity ratios. HT (height); WT (weight); BMI (Body Mass Index); Na (serum sodium); K (Serum kalium); ALB (albumin); GLB (globulin); TP (Total Protein); AST (Aspartate aminotransferase); ALT (Alanine Transaminase); TBIL (total bilirubin); DBIL (Direct Bilirubin); IBIL (Indirect Bilirubin); SCR (Serum creatinine); DRH (occurrence of diarrhea); MP (combined Montmorillonite powder); LC (combined Loperamide capsules); ER (estrogen receptor); PR (progesterone receptor).

#### 4.2.2 Data distribution and processing

**FIGURE 3:**
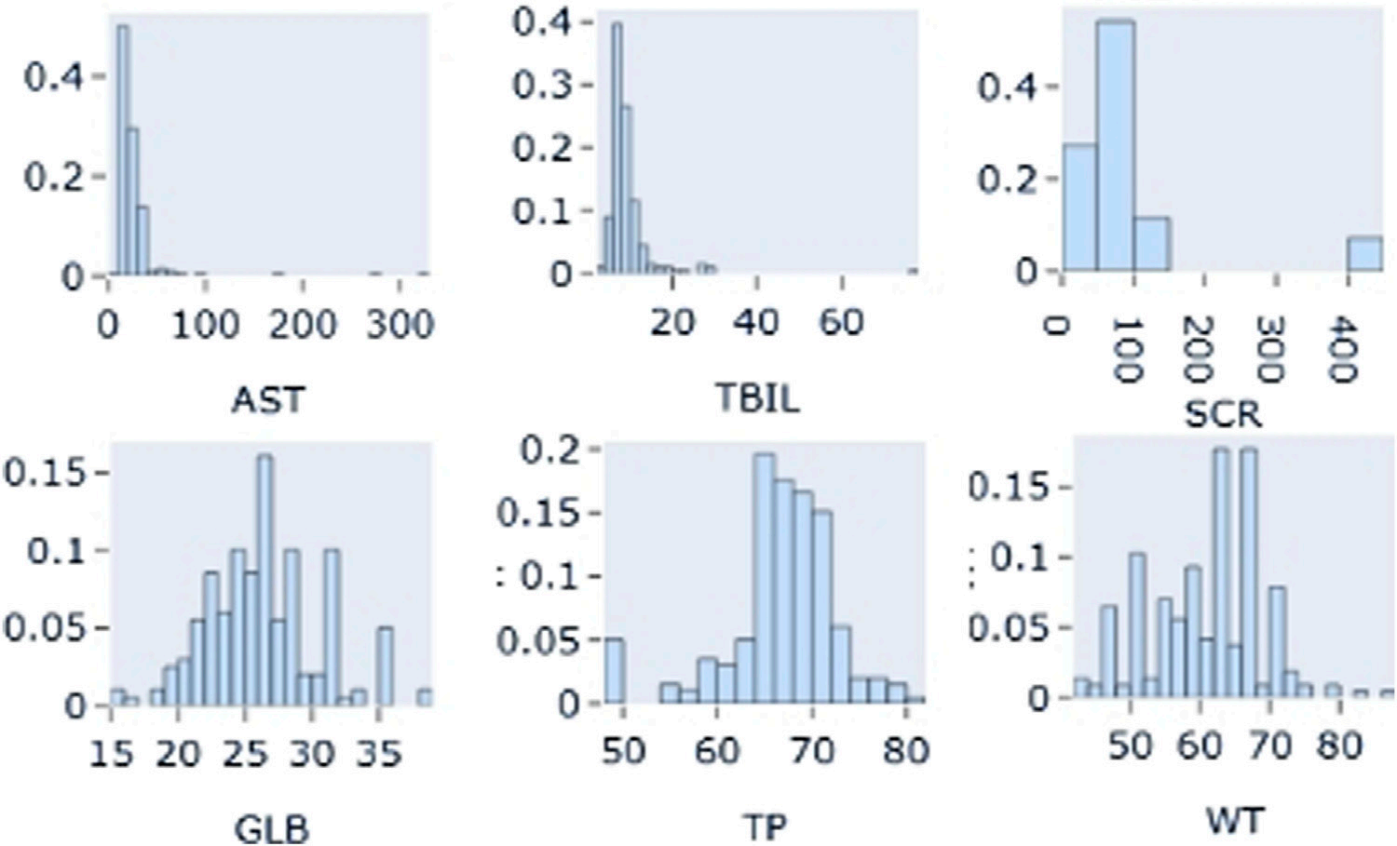
Distribution of partial continuous variables: AST: Aspartate aminotransferase; TBIL: total bilirubin; SCR: Serum creatinine; GLB: globulin; TP: Total Protein; WT: weight.

**FIGURE 4:**
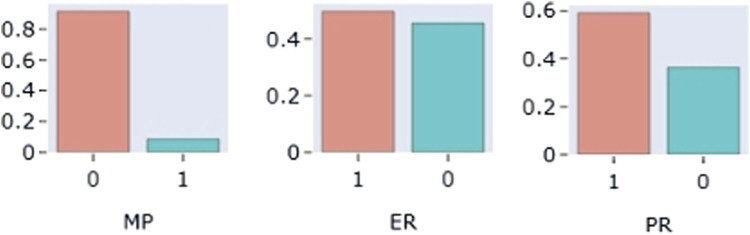
Distribution of partial categoricalvariables: MP: combined Montmorillonite powder; ER (estrogen receptor); PR (progesterone receptor).

The distribution of each covariate was plotted. The results showed that the proportion of combined use of montmorillonite powder was less than 10% of the total sample size, so this covariate was not considered. The distributions of the covariates showed that the distributions of AST, TBIL and SCR were skewed, while the distributions of the other continuous covariates were approximately normal. The distribution of some continuous variables (AST, TBIL, SCR, GLB, TP, WT) and some categorical (MP, ER, PR) covariates are shown in Figures 3, 4, respectively.

In our data, no covariate was absent for more than 20% of the patients. Every concentration in the patient sample was higher than the quantification’s lower bound. Potential outliers were defined as data whose conditional weighted residuals (CWRES) fell beyond the range of −6 to 6. In this investigation, no data were found to be outliers.

### 4.3 Population-pharmacokinetic model

#### 4.3.1 Base model

**FIGURE 5:**
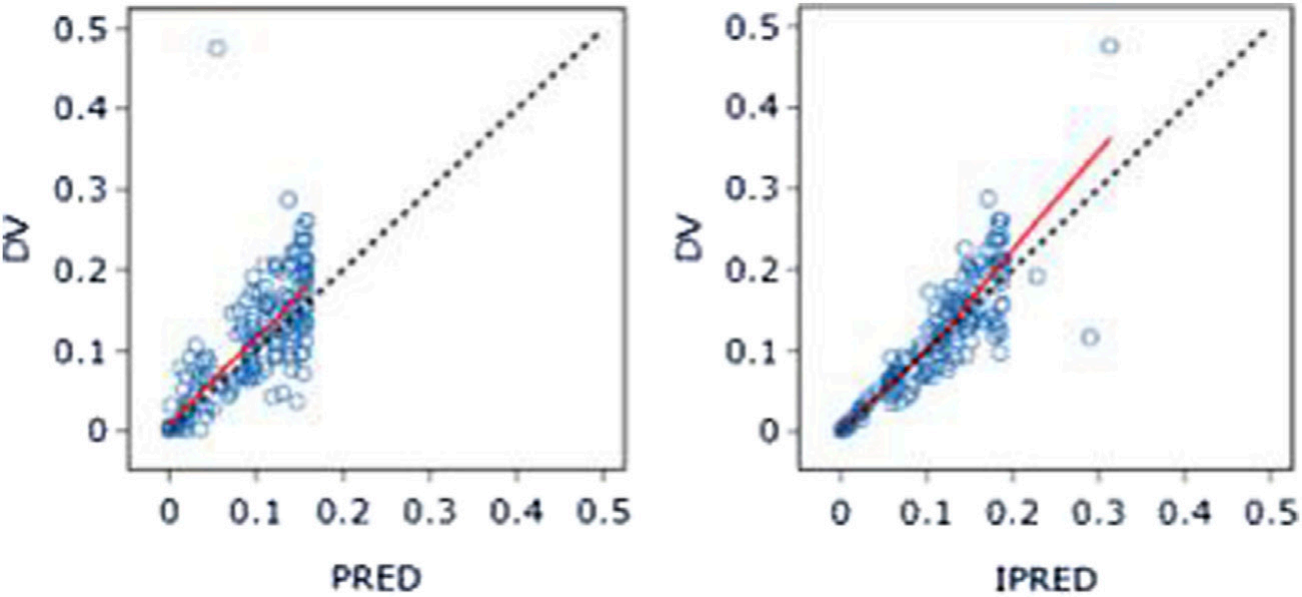
DV-PRED (left) and DV-IPRED (right)ofthebasemodel: The red line represents the locally weighted scatterplot smoothing line. CWRES, conditional weighted residual errors.

**FIGURE 6:**
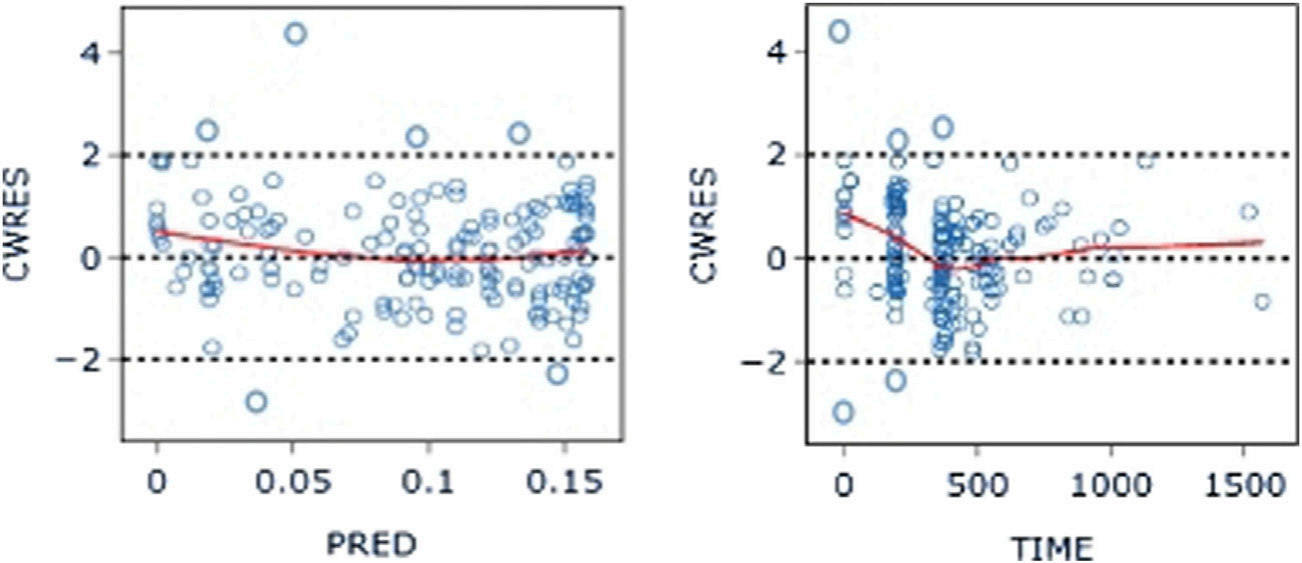
CWRES-PRED (left) and CWRES-TIME (right) of the base model: The red line represents the locally weighted scatterplot smoothing line. CWRES, conditional weighted residual errors.

The PK data exhibited an excellent GOF diagnostic plot (Figures 5, 6) and were best characterized by a one-compartment model with first-order absorption and elimination (Table 4). The absorption rate constant (Ka), apparent clearance (CL/F), and Vd/F were used to parameterize the model. In apparent clearance (CL/F) and Vd/F (Ka, FIXED), the BSV was integrated. In the studies, a proportional error model was adopted for RUV.

**TABLE 4:** Population pharmacokinetic parameter estimates of nalbuphine of the basic model.

Parameter	Estimates	RSE (%)	SHRINKSD (%)
Structural model parameter			
CL/F (L/h)	127	14.1	—
V/F(L)	3,900	26.1	—
KA (h-1)	0.357	FIXED	—
Between-subject variability			
BSV-CL (%CV)	50.3	28.9	27.3
BSV-V (%CV)	95.1	23.6	23.2
Residual unexplained variability			
ERR-1 (%CV)	28.6	5.9	15.9

CL/F,apparent clearance (L/h); TP, on CL/F, influence of total protein onapparent clearance; Vd/F, apparentvolume of distribution(L); KA, absorption rate constant; BSV-CL, between-subject variability of clearance; BSV-V, between-subject variability of volume of distribution; ERR-1, Residual Variation 1.

#### 4.3.2 Covariate model

**FIGURE 7:**
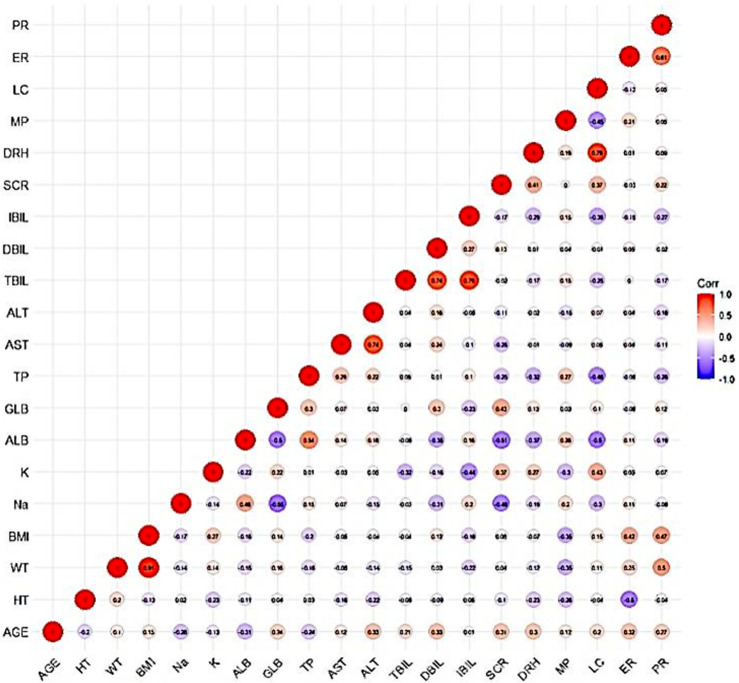
Correlation diagram between covariates: The darker color of the circle indicates a stronger correlation between the covariates. Secondly, an R-value greater than 0.7 within the circle indicates strong correlation between the covariates.

A graphical exploratory study showed that there was a correlation between the BMI and WT, a correlation between ALT and AST (R > 0.7), and a correlation between DBIL and IBIL and TBIL. WT, TBIL, and TP were thus chosen as confounders for additional investigation. Figure 7 displays a correlation diagram between the covariates. Ultimately, stepwise regression analysis was performed using a total of 15 covariates in this investigation.

The effects of TP and HT were included in the forward inclusion stage, and HT was removed in the ensuing backward elimination step. Table 5 provides the main procedures for creating the base and covariate models.

**TABLE 5:** The stepwise process of building PPK model of pyrotinib

Model no.	Model description	OFV	ΔOFV	P值
Forward inclusion				
Base model		−837.585		
1	TP-CL	−849.969	−12.384	P<0.05
2	1+HT-V	−855.245	−5.276	P<0.05
3	2+HT-CL	−859.140	−3.895	P<0.05
full covariate model		−859.140		
Backward elimination				
4	3-HT-CL	−855.245	3.895	P> 0.001
5	4-HT-V	−849.969	5.276	P> 0.001
6	5-TP-CL	−837.585	12.384	P<0.001

The final population parameter estimates for CL/F, Vd/F, and Ka are described by the following equations:

$$CL/F\left(L/h\right)=88.8\times e^{\left(TP÷67.2\right)\times 0.376}$$

$$V/F\left(L\right)=3940$$

$$KA\left(h^{-1}\right)=0.357FIXED$$

The parameters of the final model are shown in Table 6. All shrinkages of BSV and RUV were less than 30%, suggesting a reliable estimate of each parameter. The limitations of the data information (only 4 patients had data for multiple absorption phase time points, and the rest had data for only a single absorption phase) resulted in a large bias in the KA estimates, so the final estimates of KA based on the published pyrotinib PPK model were fixed to avoid unreliable parameter estimates. A sensitivity analysis needs to be performed on the final model KA values, as detailed in Table. 6. When KA = 0.357 h^−1^, the typical values of the parameters, RSE% and OFV values of the model did not change from the final model data, indicating that fixing the KA to 0.357 h^−1^ does not affect the values of the parameters, the accuracy of the estimation and the model fit.

**TABLE 6:** Sensitivity analysis of pyrotinib KA in final model.

KA (h-1)	OFV	CL/F (L/h)	TP-CL/F	V/F(L)	BSV-1 (%CV)	BSV-2 (%CV)	ERR-1 (%CV)
Finalmodel	−849.969	88	0.376	3,940	0.515	0.965	0.270
KA = 0.10	−839.288	108	0.128	2,380	0.457	1.25	0.276
KA = 0.30	−850.167	89.5	0.346	3,630	0.515	0.981	0.267
KA = 0.35	−850.077	88.9	0.373	3,900	0.515	0.966	0.269
KA = 0.357	−849.969	88.8	0.376	3,940	0.515	0.965	0.270
KA = 0.40	−848.965	88.7	0.393	4150	0.515	0.956	0.272
KA = 0.45	−847.312	88.9	0.407	4370	0.515	0.948	0.276
KA = 0.50	−847.054	89.0	0.409	4400	0.515	0.947	0.276

#### 4.3.3 Model evaluation

**FIGURE 8:**
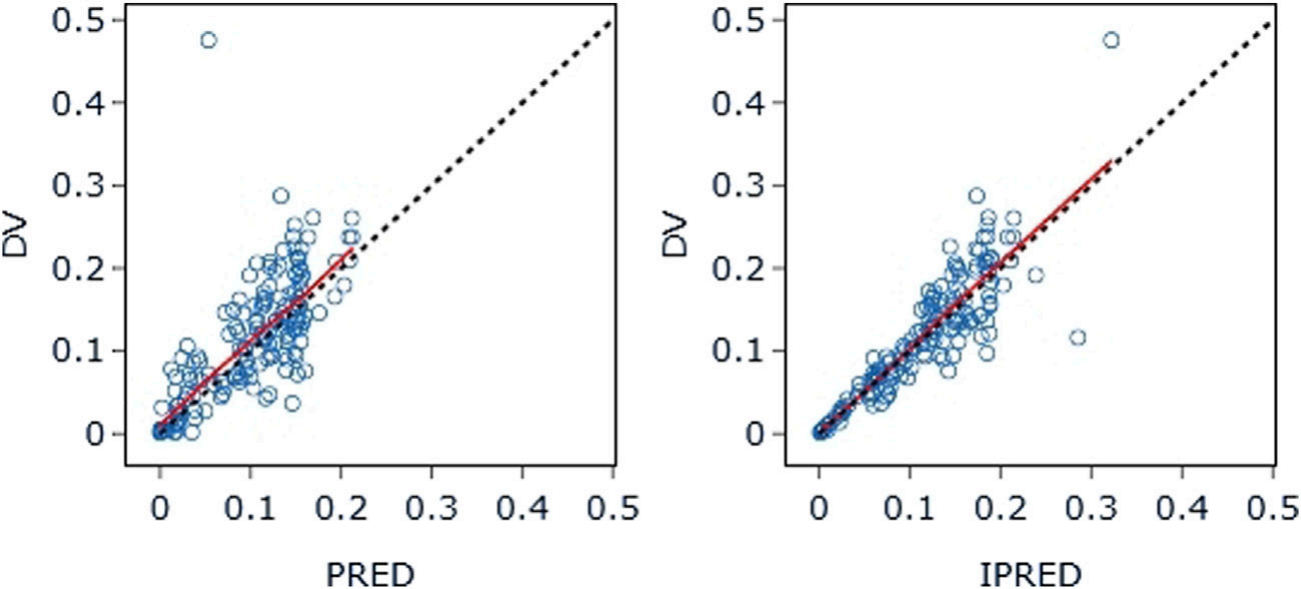
DV-PRED (left) and DV-IPRED (right) of the final model: The red line represents the locally weighted scatterplot smoothing line. CWRES, conditional weighted residual errors.

**FIGURE 9:**
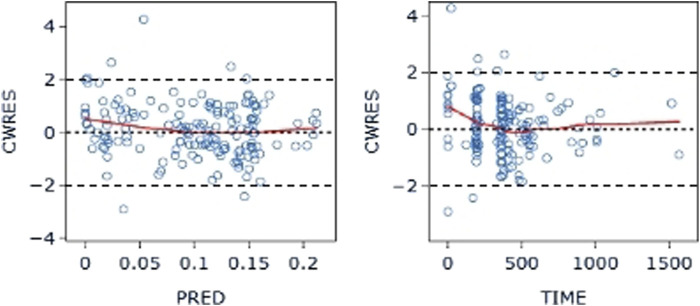
CWRES-PRED (left) and CWRES-TIME (right) of the final model: The red line represents the locally weighted scatterplot smoothing line. CWRES, conditional weighted residual errors.

The final model’s GOF diagnostic plots (Figures 8, 9) demonstrated sufficient agreement between observed and anticipated values of pyrotinib plasma concentrations, showing no signs of model bias across a broad concentration range. The scatterplots of CWRES with time and CWRES versus expectations did not show any clear trends, indicating that the final model sufficiently captured the PK of pyrotinib. Using non-parametric bootstrapping, the resulting model was resampled 1,000 times, and 915 of the calculations were able to properly show the reduction phase. The final model parameter estimates were in agreement with the bootstrapped median, 2.5th and 97.5th percentile values for each parameter (Table 7).

**TABLE 7:** Parameter estimates of pyrotinib in the final model and bootstrap evaluation.

Parameter	Final model			Bootstrap		Relative bias (%)
	Estimates	RSE%	SHRINKSD (%)	Median	2.5th–97.5th percentile	
Structural model parameter						
CL/F (L/h)	88.8	15.1	—	82.8	36.4∼217.75	−6.7
TP -CL/F	0.376	18.0	—	0.373	−0.481∼1.272	−0.8
V/F(L)	3,940	25.8	—	3,770	2,538∼6476	−4.3
KA (h-1)	0.357	FIXED	—	—	—	—
Between-subject variability						
BSV-CL (%CV)	51.5	27.3	26.5	49.5	11.9∼75.0	−3.9
BSV-V (%CV)	96.5	22.6	23.2	92.5	47.0∼29.3	−4.1
Residual unexplained variability						
ERR-1 (%CV)	27.0	6.6	16.5	27.0	24.0∼36.7	0

CL/F,apparent clearance (L/h); TP, on CL/F, influence of total protein onapparent clearance; Vd/F, apparentvolume of distribution(L); KA, absorption rate constant; BSV-CL, between-subject variability of clearance; BSV-V, between-subject variability of volume of distribution; ERR-1, Residual Variation 1.

**FIGURE 10:**
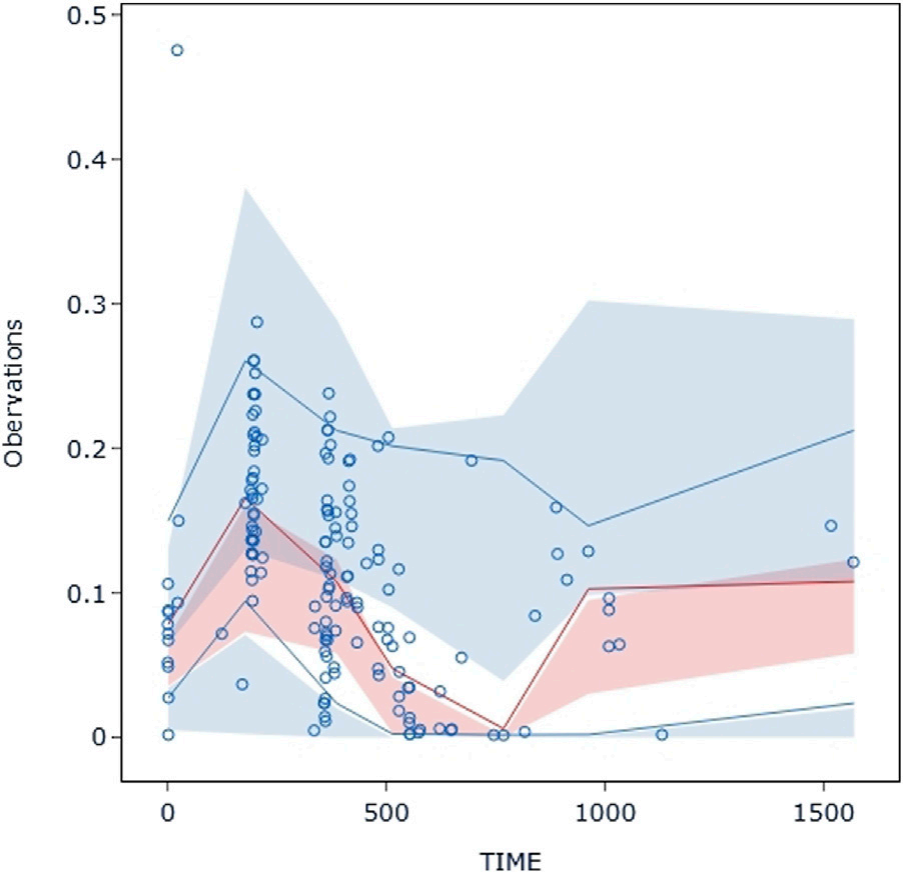
Visual predictive check: Dots represent the observed data. Solid lines represent the 5th, 50th, and 95th percentiles of the observed data. Shaded areas represent nonparametric 95% confidence intervals for the 5th, 50th, and 95th percentiles of the corresponding model-predicted percentiles.

The VPC evaluation, as shown in Figure 10, indicated that the final model correctly captured the concentration-time profile’s trend. The VPC plots overall demonstrated a fair predictive ability of the final model, with the median, 5th, and 95th percentile of the simulated pyrotinib concentrations significantly overlapping with the real values.

## 5 Discussion

Currently, chromatographic methods are considered ideal for blood concentration monitoring of small molecule targeted drugs, mainly including liquid chromatography-ultraviolet (LC-UV) and LC-MS/MS methods. Considering the low blood concentration of pyrotinib in patients, the LC-MS/MS method with higher sensitivity was chosen in this study, which was easy to operate, and suitable for monitoring the blood concentration of pyrotinib. Protein precipitation method was applied for plasma sample preparation of pyrotinib in this experiment, the commonly used organic solvents, such as methanol, acetonitrile and so on, were investigated as precipitant. This method was simple in operation, low in cost. And the method was able to quantify pyrotinib over a wider linear range (1–1,000 ng/mL).

For mass spectrometry ionization, isotope-labeled internal standards have the advantage of reducing matrix effects and fluctuations brought on by sample preparation. But we also took into account the high cost of isotope-labeled internal standards and whether or not they are stable enough to be stored for the purposes of concentration determination. As an internal benchmark for this investigation, we employed apatinib, which shares structural similarities with the medication under test. Throughout the investigation, we discovered that apatinib’s chromatographic characteristics were stable in these circumstances and had good chromatographic peaks (the batch’s RSD was less than 5%), which might satisfy the requirements of the analysis. Our results supported the use of apatinib as an effective internal standard, and the literature (Su et al., 2021) currently in publication also supports the substitution of structural analogues for isotope-labeled internal standards. In this study, 158 blood samples from 50 HER2-positive breast cancer patients were determined and included for pyrotinib PPK modeling. PK data were best described using a one-compartment model with first-order absorption and elimination, incorporating a covariate, total serum protein. This covariate had a limited effect on the PK parameters of pyrotinib, and therefore no dose adjustment of pyrotinib is recommended. Pyrotinib is mainly catalyzed by CYP3A4 enzymes in the liver (Meng et al., 2019). Total serum protein (TP) is an important indicator of liver function tests, including two types of albumin and globulin. Decreased TP clinically suggests that the synthesizing function of the liver may be impaired and is commonly associated with hepatocellular damage such as severe liver disease and liver failure. In addition, when the patient has a chronic wasting disease such as hyperthyroidism, the TP level decreases due to increased consumption.

Liver function abnormalities are one of the common adverse effects of pyrotinib. A study reported that the incidence of liver function abnormalities in pyrotinib combined with capecitabine in HER2-positive recurrent or metastatic breast cancer was 35.68% (71/199), with a predominance of grades 1–2, and the incidence of grade 3 liver function abnormalities was 2.51% (5/199) (Ma et al., 2019; Xu et al., 2021). The precautions in the leaflet for pyrotinib maleate tablets set out that liver function should be checked before starting pyrotinib therapy, and that liver function markers should be monitored at least once every two cycles (6 weeks) during the course of treatment, and the frequency of monitoring should be increased in the event of abnormalities. Treatment should be discontinued if severe liver function abnormalities were noted. Moderate to severe hepatic insufficiency may be at risk of hepatotoxicity and is not recommended. The serum total protein level reflects to some extent the status of the patient’s liver function, and the final model suggests that pyrotinib clearance was reduced in patients with low serum total protein. Therefore, in clinical practice, closely attention needs to be paid to the dosage of medication as well as to the monitoring of liver function in this group of patients in order to prevent the occurrence of adverse reactions.

Data from the Pyrotinib maleate tablets specification showed that pyrotinib was administered in combination with capecitabine to breast cancer patients, the mean clearance of 400 mg pyrotinib per day at steady state was CLss/F = 141 L/h, and the mean elimination half-life (t1/2) was 18.2 h. This value was close to the estimate of clearance (127 L/h) from the pyrotinib PPK model developed by Wen et al. (2021) based on data from two large phase I clinical trials. However, it was somewhat different from the estimated value of 88.8 L/h for the final model CL/F in this study. Possibly due to the fact that the sample data for this study were derived from a clinical real-world dosing population, and the dosages of some patients were dynamically changing (the modeling dataset contained three different dosages of 240 mg, 320 mg, and 400 mg). In addition, the final modeled clearance incorporates the effect of inter individual differences in TP on clearance. This suggests that when the TP level of patients decreases, the clearance rate of pyrotinib may decrease due to abnormal liver function, so it is necessary to pay attention to monitoring the liver function of patients at this time. At the same time, patients with abnormal albumin should be careful to take pyrroltinib, and clinical workers should pay close attention to the clinical manifestations of patients after taking it to prevent the occurrence of other serious adverse events, such as diarrhea.

The instructions for pyrotinib maleate tablets indicate that the mean apparent volume of distribution Vss/F = 4,200 L at steady state for 400 mg of pyrotinib per day when administered in combination with capecitabine for the treatment of breast cancer patients. The formula for the final PPK model developed by Wen et al. (2021) showed $V/F=2270\times {\left(AGE/47\right)}^{0.654}\times {\left(TP/72.8\right)}^{-1.94}$ , which incorporated the effects of inter-individual differences in the effect of AGE and TP on apparent distribution volume. The estimated final model V/F for pyrotinib PPK in this study was 3,940 L, and they were in comparable agreement.

It was reported that the combination of montelukast decreases the bioavailability of pyrotinib (Wen et al., 2021). However, less than 10% of the population in this study combined with montelukast to be included in the examination of covariates, pending further expansion of the sample size and optimization of the model in the future.

Second, the final model did not include the occurrence of diarrhea as an independent covariate affecting the PPK of pyrotinib, suggesting that diarrhea had a small effect on its PK. However, considering that diarrhea may also hinder drug absorption in the gastrointestinal tract (Wen et al., 2021), a confounding bias in the results cannot be excluded. The occurrence of diarrhea was included in the analysis as a dichotomous variable rather than a multicategorical covariate because the severity grading of diarrhea could not be accurately obtained for the patients in this study. Further research is needed to explore this issue in the future.

## 6 Conclusion

In this study, a simple, reproducible and accurate UPLC-MS/MS method was developed for the determination of pyrotinib concentration in humans. The results of population pharmacokinetic modeling of pyrotinib in patients with HER2-positive breast cancer suggest that clinical medical staff should pay attention to the liver function of patients with abnormal serum total protein and be alert to the occurrence of hepatotoxicity. To provide clinical medication guidance for pyrotinib, the future researche could incorporate PPK/PD modeling technology with real-world sample size expansion to optimize the current model and simulate patient dosage of pyroyinib with varying liver function grade.

## Data Availability

The raw data supporting the conclusion of this article will be made available by the authors, without undue reservation.
